# Current Rehabilitation Practices of Physiotherapists in Intensive Care Units in the UAE: A National Survey

**DOI:** 10.12688/f1000research.159853.1

**Published:** 2025-01-21

**Authors:** Monia Ashraf Megahed, Gopala Krishna Alaparthi, Emad A. Aboelnasr, Amira Hassan Bekhet, Kalyana Chakravarthy Bairapareddy, Heba Hijazi, Alham Al-Sharman, Fatma A. Hegazy

**Affiliations:** 1 Department of Physiotherapy, University of Sharjah College of Health Sciences, Sharjah, Sharjah, United Arab Emirates; 2 Department of Health Professions, Faculty of Health and Education, Manchester Metropolitan University, Manchester, England, UK; 3 Cairo University Faculty of Physical Therapy, Ad Doqi, Giza Governorate, Egypt; 4 Department of Health Care Management, University of Sharjah College of Health Sciences, Sharjah, Sharjah, United Arab Emirates; 5 Department of Health Management and Policy, Jordan University of Science and Technology Faculty of Medicine, Irbid, Irbid Governorate, Jordan; 6 Department of Rehabilitation Sciences, , Physiotherapy, Jordan University of Science and Technology, Irbid, Irbid Governorate, Jordan; 7 Neuro musculoskeletal Rehabilitation Research Group, Research Institute of Medical and Health Science, University of Sharjah, Sharjah, Sharjah, United Arab Emirates

**Keywords:** ICU Rehabilitation, Physiotherapy Practices, National Survey

## Abstract

**Background:**

Intensive care units (ICUs) are essential for patient recovery, but prolonged stays often result in complications like reduced mobility and muscle weakness.

**Aims:**

This study examines current ICU rehabilitation practices in the United Arab Emirates (UAE) through a web-based cross-sectional survey involving 80 physiotherapists from both public and private sectors.

**Methods:**

The questionnaire, validated by experts with a Cronbach’s alpha of 0.84, explored various rehabilitation strategies. Most participants held bachelor’s degrees and had 2-5 years of experience.

**Results:**

Over 96% required physician referrals for ICU physiotherapy sessions, and 75% noted that hospitals provided development programs to enhance skills. Respiratory therapy, joint mobilization, and electrical stimulation were the most commonly used interventions, while massage, continuous passive motion machines, and taping were less frequently applied. Among neonatal ICU patients, 85.7% of physiotherapists regularly performed passive range of motion exercises, and 69% consistently involved parents in the treatment plan. The results indicate a variety of practices among ICU physiotherapists in the UAE, with no clear adherence to standardized protocols. This lack of consistency may negatively affect patient care quality.

**Conclusion:**

The study underscores the importance of implementing standardized rehabilitation protocols and enhancing patient education to improve outcomes in ICU settings.

## Introduction

Intensive care Units (ICUs) are life-saving rooms that have increased the survival rates of several patient populations with various medical conditions ( Kosson et al. 2021). However, ICU admission and the nature of life-sustaining interventions have created several complications ( Bassford 2017). For example, prolonged admission to the ICU could lead to physical in-activity and pressure ulcers which potentially cause muscular atrophy, generalized weakness sepsis, infections, and deep vein thrombosis. Secondly, long-term dependency on mechanical ventilation could cause diaphragmatic weakness and which is considered one of the most life threatening ICU complications among adult patients ( Kosson et al. 2021). That is why, physical therapy and early rehabilitation is essential in ICU to prevent and treat the physical and neuropsychological consequences of ICU stay which could impede the return to normal functioning ( Kosson et al. 2021).

Hospitals in the UAE offer a variety of specialized ICUs designed to address different patient needs based on medical conditions and the level of care required. These include Medical ICUs for severe non-surgical conditions, Surgical ICUs for post-surgery recovery, Cardiac Care Units for critical heart issues, and specialized units like Neonatal ICUs for newborns, Pediatric ICUs for children, Neuro-ICUs for brain and nervous system conditions, Trauma ICUs, Burn Units, Respiratory ICUs, and Transplant ICUs ( Latif et al. 2015). The range and capacity of these units vary by hospital, providing comprehensive care tailored to specific patient needs.

In the ICU, physiotherapists aim to preserve or improve physical function, muscle strength, exercise tolerance and physical activities ( Bassford 2017). Physical therapy interventions include positioning, education, manual and ventilator hyperinflation, weaning from mechanical ventilation, non-invasive ventilation, percussion, vibration, suctioning, respiratory muscle strengthening, breathing exercises and mobilization ( Pascale et al. 2022). The former interventions boost muscle strength, physical functioning and quality of life as well as, delirium ( Kanejima et al. 2020; Eggmann et al. 2021; Pascale et al. 2022). In clinical practice, in addition to safety issues, there are several clinical barriers for the implementation of physical therapy for critically ill patients in the ICU. Recent literature report that lack of staff and time, potential risks of airway dislodgement, and the dislocation of intravenous and arterial lines are the common barriers for early rehabilitation in the ICU ( Anekwe et al. 2020; Cuthbertson et al. 2020; Morrow 2021).

Several studies have reported the rehabilitation practice of physiotherapists in various countries. For instance, a UK study was conducted to investigate the use of physiotherapists of evidence-based practice (EBP) guidelines in ICU ( Stockley et al. 2010). In Australia, another study was conducted in 2010 to investigate the efficacy of passive limb range of motion on adults in ICU ( Wiles and Stiller 2010).

In the UAE, the number of practicing physiotherapists is 3,567, of them only 314 members are registered in the UAE wing of World Confederation of Physiotherapy (WCPT) In 2021, a study was conducted to investigate the attitude of UAE physiotherapists toward the use of EBP guidelines ( AlKetbi et al. 2021). It has been reported that the attitude towards EBP was related to the knowledge of EBP and the perception of barriers. Moreover, physiotherapists prefer to use their own experience, published textbooks and research articles to apply EBP guidelines ( AlKetbi et al. 2021). The previous results have reported a variation of practice among physiotherapists in ICU which could lead negatively the patients and deteriorate their physical and cardiopulmonary functions. That is why we formulated a questionnaire to investigate the current rehabilitation practices of UAE physiotherapists in ICU.

The United Arab Emirates (UAE) currently has 3,567 practicing physiotherapists, yet only 314 are registered members of the UAE chapter of the World Confederation for Physical Therapy (WCPT) ( AlKetbi et al. 2021). This discrepancy raises concerns about the alignment of local physiotherapy practices with global standards. In 2021, a study examining the attitudes of UAE physiotherapists toward Evidence-Based Practice (EBP) guidelines highlighted a significant correlation between attitudes toward EBP and both knowledge of EBP and perceived barriers to its implementation ( AlKetbi et al. 2021). This study also revealed that many physiotherapists rely on personal experience, textbooks, and research articles as primary sources of knowledge for applying EBP, rather than formally adhering to standardized guidelines ( AlKetbi et al. 2021). Additionally, there is evidence of practice variation among physiotherapists working in ICUs, which could have detrimental effects on patients’ physical and cardiopulmonary outcomes ( Wiles and Stiller 2010). Inconsistent rehabilitation approaches in such critical settings may lead to suboptimal recovery, potentially prolonging ICU stays and increasing healthcare costs.

Given the importance of standardized, evidence-based rehabilitation practices in improving patient outcomes, there is a need to investigate the current ICU rehabilitation practices among physiotherapists in the UAE. Ultimately, this research may potentially contribute to improving rehabilitation care in ICUs, ensuring that UAE physiotherapists are better equipped to follow consistent, evidence-based protocols that benefit patient recovery and overall health outcomes.

### Study objectives

This study aims to explore the current physiotherapy practices in ICUs across the UAE. By investigating these practices, the study aims to improve patient outcomes and elevate the quality of care provided. Additionally, the findings of this research have the potential to inform healthcare policies optimizing resource allocation and ensuring that patients receive the most effective physiotherapy services. Ultimately, this study seeks to promote collaboration and innovation in critical care physiotherapy, contributing to the advancement of healthcare practices in the UAE.

## Methods

### Study design and participants

This prospective cross-sectional study was conducted in the UAE and included physiotherapists registered with the Emirates Physiotherapist Society (EPS) from all emirates. Eligible participants were physiotherapists working in private or public UAE hospitals, holding at least a bachelor’s degree, and having a minimum of one year of experience. Physiotherapists not specialized in ICU rehabilitation were excluded from the study.

Ethical approval was obtained from the Research Ethics Committee of the University of Sharjah. A list of hospitals that offer physiotherapy services was collected from the EPS, and the emails of the registered physiotherapists were retrieved from the EPS website. A cover letter explaining the study’s purpose, along with the questionnaire, was then emailed to the physiotherapists. Participants received a hyperlink to the informed consent form, and those who consented to participate were granted access to the questionnaire. Data collection started on the 19 ^th^ of September 2022 and completed on the 2 ^nd^ of December 2023.

### Sample size calculation

This study included 80 physiotherapists from various hospitals in the UAE. This sample size was calculated depending on the proportion (90%) stated in a previous study addressing the research question ( Çakmak et al. 2019). The appropriate sample size with sufficient power of 80% (alpha = 0.05) was calculated by the Epi Info, a program developed by the Centers for Disease Control and Prevention ( Centers for Disease Control and Prevention 2022).

### Survey development

The study aimed to review the current practices of physiotherapists in ICUs in the UAE. A literature review was conducted to identify gaps in knowledge and inform survey questions. Questions were developed based on the literature review, covering participant demographics, types of interventions used, session frequency and duration, and challenges faced by physiotherapists in delivering care. The survey was created with a mix of open-ended and close-ended Likert questions for easy administration and analysis. The questions were kept simple and short to be familiar to the participants. The survey was initially done in English before being transferred to an online platform using Google Forms. The data collected was then downloaded into Excel for further analysis.

The survey was pre-tested with a small group of physiotherapists to make sure it was clear, relevant, and easy to understand. The feedback from the testing was used to improve the survey, ensuring that the questions were clear and accurately captured the information needed from participants. This process helped fine-tune the survey before it was given to a larger group of physiotherapists working in ICUs in the UAE.

### Questionnaire validity and reliability

To examine the questionnaire’s validity, a group of experts specialized in physiotherapy practice was invited to judge the content, construct, clarity, and relevance. After the formulation of the first draft that included 30 items gathered from the previous literature, the experts added 36 items. Afterward, the experts assessed the relevance and clarity of the questions and the significance and completeness of responses. Each item in the questionnaire was assessed by the experts on Likert scale that ranges from 1-10 where a score that ranges from 1 to 4 means irrelevant or unclear, a score that ranges from 5 to 7 means partially relevant or partially clear, and a score that ranges from 8 to 10 means highly relevant or clear. Then, they started an open online discussion with several coworkers from different emirates in the UAE to assure the understandability, completeness, plausibility, and management of the instrument.

For reliability analysis, a different group of ICU physical therapists in UAE were invited online to test the reliability of the questionnaire. Thirty physical therapists completed the process of pilot testing followed by the calculation of Cronbach’s alpha which is a way of assessing reliability by comparing the amount of shared variance, or covariance, among the items making up an instrument to the amount of overall variance. Finally, Cronbach’s alpha value of 0.843 was inducted which infers a good reliability.

### Statistical analysis

Data was extracted from Google Forms and then IBM SPSS Statistics 27 was used to conduct all the descriptive statistics and analysis. Descriptive data analysis included calculating the frequencies and percentages of the participants’ demographic data. Chi square test was used to study the correlation between the different domains of the questionnaire and the demographic data.

### Ethical statement

This research project was approved by the Ethics Committee of the University of Sharjah, UAE [Certificate# REC-21-07-04-01-S]. Also, written informed consents were obtained from all participants before proceeding with the study questionnaires. All study procedures were conducted according to the ethical considerations stated by the declaration of Helsinki ( Goodyear et al. 2007). At the beginning of the questionnaire, participants were asked to read a short paragraph about the components of the questionnaire and the main objectives. After that, if they agree to participate, they must click the “agree to participate” button before proceeding to fill in the questionnaire. However, they have the option of quitting the questionnaire at any time while they are proceeding through the various aspects of the questionnaire.

## Results

### Demographic Characteristics

As shown in Table 1, a total of 80 physiotherapists participated in the study, comprising 47.5% females and 52.5% males. The age range of participants varied, with nearly two-thirds of participants (61.3%) in the age group of 25 to 34 years. Regarding the distribution across emirates, 40% of the participants were working in Dubai, 27.5% in Abu Dhabi, 25% in Sharjah, and the rest were from other emirates. In terms of the type of hospital, 47.4% of participants worked in private hospitals, 48.8% in public hospitals, and only 3.8% in university hospitals.

**Table 1.** Demographic characteristics of the overall sample (n=80).

Variables		Frequency	Percent
Gender	Female	38	47.5
	Male	42	52.5
Age	24 or below	3	3.8
	25-34	49	61.3
	35-44	23	28.8
	45-54	5	6.3
Which emirate are you currently working in?	Abu Dhabi	22	27.5
	Ajman	3	3.8
	Dubai	32	40.0
	Fujeirah	1	1.3
	Ras al-Khaimah	2	2.5
	Sharjah	20	25.0
Type of hospital	Private hospital	38	47.4
	Public hospital	39	48.8
	University hospital	3	3.8
No. of experience years as physiotherapist	0-2 years	9	11.3
	2-5 years	44	55.0
	5-10 years	15	18.8
	10-15 years	7	8.8
	15-20 years	3	3.8
	20+ years	2	2.5
No. of experience years as physiotherapist in ICU	0-2 years	20	25.0
	2-5 years	7	8.8
	5-10 years	40	50.0
	10-15 years	13	16.3
	15-20 years	20	25.0
	20+ years	7	8.8
Last earned academic degree in physiotherapy	BSc	42	52.5
	Diploma	3	3.8
	MSc	25	31.2
	PhD	10	12.5
Do you require a physician’s referral to start physiotherapy sessions in the ICU?	No	3	3.8
	Yes	77	96.3
Is there a program at the hospital where you work to develop the level of physiotherapists working in ICUs?	No	20	25.0
	Yes	60	75.0
Are the protocols for physical therapy assistance defined by a scientific team from the service in which you are part of?	No	20	25.0
	Yes	60	75.0
Does the physiotherapy service have any certification or quality seal?	No	14	17.5
	Yes	66	82.5

Participants’ years of experience as a physiotherapist ranged from 1 to over 20 years, with about 55% of the sample having physiotherapy experience between 2 and five years. Specifically, the years of experience working in ICU settings were noticeable, with 50% of participants having between 5 and 10 years of ICU experience. Regarding the last earned academic degree in physiotherapy, 52.5% of participants held a bachelor’s degree, 31.2% had a master’s degree, 12.5% held a doctoral degree, and only 3.8 had a diploma in physiotherapy.

Among the participants, the vast majority (96.3%) reported that a physician’s referral was necessary to initiate physiotherapy sessions in the ICU, while only 3.8% indicated no requirement for a referral. Additionally, 75% of physiotherapists reported the presence of a development program at their hospital aimed at enhancing the skills of physiotherapists working in ICUs.

Regarding the establishment of protocols for physical therapy assistance, 75% of participants reported that the protocols were defined by a scientific team within their service, while 25% reported otherwise. Furthermore, 82.5% of physiotherapy services had certification or quality seals indicating adherence to standardized practices.

### Physiotherapy respiratory practices

Table 2 presents the survey results of physiotherapy practices. These results provide valuable insights into the different practices among physiotherapists. Regarding the assessment of vital parameters such as heart rate, respiratory rate, and SPO _2_ pre- and post-treatment, a majority of respondents (84.1%) reported assessing these parameters always. However, a notable portion (7.2%) reported only sometimes or seldom conducting these assessments, suggesting potential variability in clinical protocols.

**Table 2.** Physiotherapy practices among the study sample.

Survey items	Always	Frequently	Sometimes	Seldom	Never
Do you assess vital parameters (Heart rate, Respiratory rate, SPO _2_) pre and post treatment?	58 (84.1%)	6 (8.7%)	4 (5.8%)	1 (1.4%)	0
Are you involved in setting ventilator parameters?	38 (55.1%)	8 (11.6%)	13 (18.8%)	1 (1.4%)	9 (13%)
Do you teach and prescribe deep breathing exercises?	51 (73.9%)	15 (21.75%)	3 (4.3%)	0	0
Do you perform vibration?	50 (72.5%)	14 (20.3)	0	0	5 (7.2%)
Do you perform suctioning?	44(63.8%)	4 (5.8%)	8(11.6%)	1 (1.4%)	12 (17.4%)
Do you perform percussion?	54 (78.3%)	11 (15.9%)	2 (2.9%)	2 (2.9%)	0
Do you perform postural drainage position?	46 (66.7%)	13 (18.8%)	9 (13%)		1 (1.4%)
Do you perform prone postural drainage position?	15 (21.7%)	13 (18.8%)	24 (34.8%)	10 (14.5%)	7 (10.1%)
Do you perform head down postural drainage position in ventilated patients?	12 (17.4%)	8 (11.6%)	25 (36.2%)	12 (17.4%)	12 (17.4%)
Do you use AMBU while performing chest physiotherapy and/suctioning?	37 (53.6%)	4 (5.8%)	11 (15.9%)	5 (7.2%)	12 (17.4%)
Are you involved in the weaning of the patient from the mechanical ventilation?	31 (44.9%)	8 (11.6%)	16 (23.2%)	7 (10.1%)	7 (10.1%)
Do you apply nebulizer before the treatment?	25 (36.2%)	13 (18.8%)	24 (34.8%)	2 (2.9%)	5 (7.2%)
Do you apply nebulizer post treatment?	34 (49.3%)	11 (15.9%)	13 (18.8%)	4 (5.8%)	7 (10.1%)
Do you make discharge recommendations for the progression of rehabilitation at home?	49 (71%)	14 (20.3%)	4 (5.8%)	0	2 (2.9%)
Do you involve and advice parents/caregivers in the treatment plan and/or discharge plan?	51 (73.9%)	14 (20.3%)	3 (4.3%)	1 (1.4%)	0

In terms of involvement in ventilator management, 55.1% of participants reported frequent participation in setting ventilator parameters, indicating a significant role in critical care settings. However, a considerable proportion (13%) reported never involvement in ventilator management, suggesting potential differences in practice patterns among physiotherapists. Similarly, while the prescription and teaching of deep breathing exercises were noted to be frequent by 73.9% of respondents, a small portion (4.3%) indicated some involvement, highlighting potential inconsistencies in patient education practices.

Respiratory therapy techniques such as vibration, suctioning, and percussion were reported to be performed with varying frequencies. For instance, 72.5% of participants reported frequent use of vibration, while 63.8% reported frequent suctioning. Additionally, the utilization of postural drainage positions varied, with approximately 66.7% of respondents performing postural drainage positions frequently.

The use of AMBU during chest physiotherapy and suctioning was reported to be frequent by more than half (53.6%) of participants. Similarly, nearly half (44.9%) of the respondents reported frequent involvement in the weaning of patients from mechanical ventilation, underscoring the integral role of physiotherapists in the critical care team.

Regarding nebulizer application, approximately 36.2% of respondents reported frequent use before treatment, while 49.3% reported frequent use post-treatment. This suggests that nebulizer therapy is commonly integrated into respiratory treatment plans, although there may be variations in timing and protocol adherence.

Lastly, the survey revealed that a majority of physiotherapists frequently make discharge recommendations for the progression of rehabilitation at home (71%) and involve/advice parents or caregivers in the treatment plan and/or discharge plan (73.9%). This emphasis on patient education and continuity of care highlights the holistic approach adopted by physiotherapists in respiratory therapy practice.

### Physiotherapy modalities and techniques

As presented in Table 3, the survey results provide comprehensive insights into the utilization of various physiotherapy modalities and techniques in the ICU setting. Among the surveyed physiotherapists, the application of passive/active range of motion exercises emerged as a common practice, with the majority (92.5%) reporting always applying these exercises. Additionally, bed mobility exercises and assistance in bed transfers were frequently implemented by 83.6% and 79.1% of respondents, respectively, highlighting the emphasis on promoting functional mobility and independence among ICU patients.

**Table 3.** Physiotherapy modalities and techniques.

Survey items	Always	Frequently	Sometimes	Seldom	Never
Do you apply passive/active range of motion exercises?	62 (92.5%)	2 (3%)	3 (4.5%)	0	0
Do you apply bed mobility exercises?	56 (83.6%)	8 (11.9%)	3 (4.5%)	0	0
Do you apply/assist in bed transfers?	53 (79.1%)	9 (13.4%)	4 (6%)	1 (1.5%)	0
Do you apply neuromuscular electrical stimulation?	38 (56.7%)	5 (7.5%)	12 (17.9%)	4 (6%)	8 (11.9%)
Do you perform stretching exercises?	39 (58.2%)	14 (20.9%)	14 (20.9%)	0	0
Is continuous passive motion (CPM) machine used in your ICU setup?	40 (59.7%)	4 (6%)	6 (9%)	5 (7.5%)	12 (17.9%)
Do you apply massage techniques?	24 (35.8%)	3 (4.5%)	23 (34.3%)	4 (6%)	13(19.4%)
Do you apply scar tissue mobilization?	22 (32.8%)	4 (6%)	28 (41.8%)	4 (6%)	9 (13.4%)
Do you apply taping?	13 (19.4%)	5 (7.5%)	26 (38.8%)	10 (14.9%)	13 (19.4%)
At discharge, do you routinely apply a walk test?	30 (44.8%)	9 (13.4%)	17 (25.4%)	6 (9%)	5 (7.5%)
Do you make discharge recommendations for the progression of rehabilitation at home?	49 (73.1%)	11 (16.4%)	6 (9%)	1 (1.5%)	0
Do you involve and advice parents/caregivers in the treatment plan and/or discharge plan?	49 (73.1%)	13 (19.4%)	4 (6%)	1 (1.5%)	0

Neuromuscular electrical stimulation, a modality used to facilitate muscle contraction and improve muscle strength, was reported to be frequently applied by 56.7% of participants. Similarly, stretching exercises were commonly performed by 58.2% of respondents, indicating the integration of flexibility training into rehabilitation protocols for ICU patients. However, continuous passive motion (CPM) machines, which aid in joint mobilization and rehabilitation, were less common, with about one-quarter of respondents reporting seldom or never using it in the ICU setting.

Massage techniques and scar tissue mobilization were reported to be moderately applied by respondents, with 35.8% and 32.8%, respectively. Taping, another therapeutic modality used to provide support and stability to muscles and joints, was reported to be frequently applied only by 19.4% of participants.

At discharge, 44.8% of respondents routinely applied a walk test, while the majority of physiotherapists (73.1%) reported routinely making discharge recommendations for the progression of rehabilitation at home.

Involving and advising parents/caregivers in the treatment and discharge plan was considered essential by the majority of respondents (73.1%). This emphasis on patient and caregiver involvement underscores the collaborative approach to patient care in the ICU, emphasizing the importance of patient education in promoting optimal outcomes.

### Utilization of physiotherapy techniques on neonates

The last part of the survey included questions about the utilization of physiotherapy techniques, specifically on neonate patients (see Table 4). The results provide valuable insights into the utilization of various physiotherapy modalities and approaches for neonates in the NICU setting. Passive range of motion exercises emerged as a commonly performed intervention, with the majority of respondents (85.7%) indicating that they always perform these exercises for neonates in the NICU.

**Table 4.** Application of physiotherapy techniques on neonates.

Survey items	Always	Frequently	Sometimes	Seldom	Never
Do you perform passive range of motion exercises for neonates in the NICU?	36 (85.7%)	4 (9.5%)	2 (4.8%)	0	0
Do you apply positioning to support alignment and movement?	30 (71.4%)	6 (14.3%)	6 (14.3%)	0	0
Do you perform therapeutic handling for neonates with movement impairments?	28 (66.7%)	6 (14.3%)	5 (11.9%)	3 (7.1%)	0
Do you give orofacial stimulation in neonates?	21 (50%)	5 (11.9%)	6 (14.3%)	10 (23.8%)	0
Do you consider hydrotherapy?	4 (9.5%)	2 (4.8%)	22 (52.4%)	4 (9.5%)	10 (23.8%)
Do you apply massage techniques for neonates?	9 (21.4%)	5 (11.9%)	15 (35.7%)	5 (11.9%)	8 (19%)
Do you apply scar tissue mobilization for neonates?	10 (23.8%)	4 (9.5%)	16 (38.1%)	4 (9.5%)	8 (19%)
Do you apply taping for neonates?	6 (14.3%)	3 (7.1%)	15 (35.7%)	5 (11.9%)	13 (31%)
Do you teach parents skin to skin holding (kangaroo mother care) for neonates?	16 (38.1%)	2 (4.8%)	15 (35.7%)	3 (7.1%)	6 (14.3%)
Do you involve and advice parents in the treatment plan for neonates?	29 (69%)	5 (11.9%)	6 (14.3%)	1 (2.4%)	1 (2.4%)

Positioning to support alignment and movement was reported to be frequently applied, with 71.4% of respondents indicating that they frequently employ positioning techniques for neonates in the NICU. Proper positioning is essential for optimizing neuromuscular development and facilitating motor skills acquisition in neonates, emphasizing the proactive approach of physiotherapists in promoting optimal positioning practices in the NICU.

A significant proportion of respondents reported therapeutic handling for neonates with movement impairments, with 66.7% indicating that they frequently perform therapeutic handling interventions. Likewise, orofacial stimulation in neonates was reported to be always considered by 50% of respondents. However, nearly one-quarter (23.8%) reported seldom use of this approach with the neonates. This practice aims to promote oral feeding skills and sensory development in neonates; however, a variability exists in using orofacial stimulation techniques among physiotherapists in the NICU setting.

Hydrotherapy, a modality involving therapeutic activities in water, was never considered by 23.8% of respondents, with only 9.5% indicating that they always consider hydrotherapy for neonates. While hydrotherapy has shown benefits in specific populations, its feasibility and safety in the NICU setting may be limited, warranting further investigation and consideration of alternative approaches.

Massage techniques and scar tissue mobilization were reported to be occasionally applied by respondents, with 21.4% and 23.8%, respectively, indicating that they always employ these interventions for neonates in the NICU. These practices may promote relaxation, circulation, and tissue healing in neonates, highlighting the importance of individualized and holistic care approaches in the NICU.

The utilization of taping for neonates was infrequent, with only 7.1% of respondents indicating that they sometimes apply taping techniques. Taping may provide support and stability to neonatal joints and muscles, suggesting potential variations in the utilization of this modality among physiotherapists in the NICU setting.

Teaching parents skin-to-skin holding, also known as kangaroo mother care, emerged as a relatively commonly practiced intervention, with 38.1% of respondents indicating that they always teach parents skin-to-skin holding for neonates. Furthermore, the involvement and advice of parents in the treatment plan for neonates were considered essential by the majority of respondents (69%).

## Discussion

This study aimed to investigate the current rehabilitation practices of physiotherapists in ICUs in the UAE. To the best of the researchers’ knowledge, this is the first study in the UAE that focuses explicitly on physiotherapists’ practices in the ICUs. The findings of this study shed light on various demographic characteristics, professional experiences, and institutional factors among physiotherapists working in ICU settings in the UAE.

### The demographic findings

The gender distribution among participants revealed a reasonably balanced representation, with slightly more male physiotherapists than females. This balance could reflect the increasing gender diversity within the field of ICU physiotherapy, which has traditionally been dominated by females ( Ou et al. 2022). However, further exploration into gender dynamics within different practice settings and their potential impact on patient care outcomes could help understand why female physiotherapists are more dominant in ICUs than their male counterparts.

The age distribution of participants highlighted a significant proportion of physiotherapists belonging to the younger age groups, particularly between 25 and 34 years old. A similar age range was also reported in Singapore ( Ou et al. 2022) and Brazil ( Matilde et al. 2018), indicating that relatively younger physiotherapists are in the ICU workplace. This demographic trend could signify a generational shift within the profession, with implications for workforce dynamics, skillsets, and career trajectories. Future studies could investigate the factors influencing career choices and job satisfaction among younger physiotherapists, as well as their perceptions of working in ICU settings.

Regarding the distribution across emirates and types of hospitals, the predominance of physiotherapists working in Dubai and private and public hospitals is noteworthy. This distribution reflects the urban concentration of healthcare facilities in Dubai and the prominence of private healthcare providers in the UAE. Understanding the geographic distribution of physiotherapists and healthcare resources is crucial for workforce planning and ensuring equitable access to rehabilitation services across different regions.

The years of experience among participants indicate a diverse range of professional backgrounds, with a considerable proportion having substantial experience in both general physiotherapy practice and specifically in ICU settings. A study in Saudi Arabia reported half of the participants had over ten years of experience in the ICU ( Alqahtani et al. 2020). In our study, however, slightly over half of the participants (55%) had experience between two and five years, reflecting the younger physiotherapists working in the ICUs in the UAE. This is an interesting demographic finding as the depth of experience could contribute to enhanced clinical expertise and proficiency in managing complex cases in critical care environments ( Viloria et al. 2023). It also underscores the need for ongoing professional development and training programs to support the continuous learning needs of physiotherapists throughout their careers.

The requirement for a physician’s referral to initiate physiotherapy sessions in the ICU is a notable finding, with the vast majority of participants indicating its necessity. Similar results have been documented in Saudi Arabia ( Alqahtani et al. 2020), Brazil ( Matilde et al. 2018), and Nepal ( Baidya et al. 2016). However, there are variations in physician’s referral requirements based on the geographic location and the nature of the healthcare system. For example, in Canada, physiotherapy practice is a primary healthcare service; therefore, physician referrals are not required in most cases ( Bath et al. 2018). In the middle eastern countries, physician’s referrals are required for physiotherapy services. Generally, the referral requirement may reflect the collaborative nature of healthcare delivery in ICU settings in the UAE, where interdisciplinary teamwork is essential for optimizing patient outcomes. However, further exploration of the referral process, its efficiency, and potential barriers to timely access to physiotherapy services could be warranted to improve care coordination and patient access.

Finally, the high prevalence of certification or quality seals among physiotherapy services indicates a commitment to adherence to standardized practices and quality assurance within healthcare institutions. These certifications serve as indicators of excellence and may enhance patient confidence in the quality of care provided by physiotherapists in ICU settings. Collectively, these findings provide valuable insights into the demographic characteristics, professional experiences, and institutional factors shaping ICU physiotherapy practice in the UAE. By understanding these dynamics, healthcare stakeholders can identify areas for improvement, implement targeted interventions, and ultimately enhance the quality and effectiveness of physiotherapy services in critical care settings. Future research endeavours could further explore the impact of these factors on patient outcomes, healthcare delivery models, and workforce development strategies.

### Physiotherapy practices in the ICU

In terms of physiotherapists’ practices, the results offer valuable insights into the diverse practices among participants, highlighting both areas of consistency and potential variations in clinical protocols. Firstly, assessing vital parameters such as heart rate, respiratory rate, and SPO _2_ pre- and post-treatment emerged as a common practice among most respondents, with 84.1% reporting these parameters always. This finding highlights the importance of thorough patient monitoring in respiratory care, facilitating informed decision-making and timely interventions. Previous research consistently recommended assessing vital signs before applying clinical interventions in the ICU ( Sapra et al. 2024). However, a notable portion (7.2%) reporting sometimes or seldom conducting these assessments suggests the need for standardization and adherence to clinical guidelines to ensure comprehensive patient evaluation across all practice settings.

Involvement in ventilator management emerged as another significant aspect of respiratory therapy practice, with over half (55.1%) of participants reporting frequent participation in setting ventilator parameters. This highlights the pivotal role of physiotherapists in critical care settings, where optimizing ventilator settings is crucial for patient outcomes. However, the proportion of respondents (13%) reporting never involvement in ventilator management signals potential disparities in practice patterns, which could impact the consistency and quality of care delivered in these settings. Physiotherapists in the ICU often administer treatments to sedated and mechanically ventilated patients with pneumonia to enhance airway clearance, optimize pulmonary mechanics, enhance gas exchange, and support weaning from mechanical ventilation and functional recovery ( van der Lee et al. 2021). Nevertheless, there is inconsistency in clinical practice regarding the specifics of physiotherapy intervention, including type, duration, and frequency, and there is currently no established standard of practice ( Lee et al. 2017). It is evident that standardizing protocols and promoting interdisciplinary collaboration may help address these variations and ensure optimal patient care.

The survey results on the utilization of various physiotherapy modalities and techniques within the ICU setting revealed interesting trends. For example, passive/active range of motion exercises emerged as a predominant intervention, with an overwhelming majority (92.5%) of respondents reporting always applying these exercises. Although previous reports suggested that these exercises are performed only by 15.4% of physiotherapists in the ICU ( Reid et al. 2018), the dominant passive/active range of motion exercises in our study may indicate paradigm shift towards manual techniques over other modalities in the UAE. This also underscores the recognized importance of maintaining joint mobility and preventing contractures among ICU patients, contributing to their overall functional recovery and rehabilitation ( Rahiminezhad et al. 2022).

A considerable proportion of respondents reported the utilization of neuromuscular electrical stimulation (NMES) and stretching exercises to improve muscle strength and flexibility (56.7% and 58.2%, respectively). Research shows that critically ill patients who underwent NMES in the ICU showed less reduction in muscle thickness and better functional outcomes ( García-Pérez-de-Sevilla and Sánchez-Pinto Pinto 2023). However, it did not seem to be effective in older patients (>75 years) ( Nonoyama et al. 2022), which may explain why only half of the participants in this current study applied the NMES. Moreover, the relatively lower utilization of continuous passive motion (CPM) machines, reported by about one-quarter of respondents, suggests potential opportunities for further exploration of its benefits and integration into ICU rehabilitation protocols.

In contrast, hydrotherapy, a modality involving therapeutic activities in water, was seldom considered by a notable proportion of respondents, reflecting potential limitations and challenges in implementing hydrotherapy in the ICU setting. The limited utilization of hydrotherapy may be attributed to concerns regarding safety, feasibility, and logistical constraints in the ICU environment, warranting further investigation and consideration of alternative approaches. To ensure patient safety, when implementing hydrotherapy, several factors should be taken into account, such as staff experience, airway and ventilation management, and patient readiness for the procedure ( Felten-Barentsz et al. 2015; Wegner et al. 2017).

At discharge, while a walk test was routinely applied by less than half of the respondents (44.8%), the majority (73.1%) reported routinely making discharge recommendations for the progression of rehabilitation at home. This underscores the proactive approach of physiotherapists in planning for patients’ post-discharge rehabilitation needs, aiming to facilitate a smooth transition from the ICU to the home environment. Furthermore, the majority of respondents (73.1%) emphasized the importance of involving and advising parents/caregivers in the treatment and discharge plan. This collaborative approach underscores the recognition of the crucial role that caregivers play in supporting patients’ recovery and rehabilitation at home, emphasizing the importance of patient education and continuity of care beyond the hospital setting.

Overall, the diverse range of physiotherapy modalities and techniques reported in this study reflect a holistic approach to patient care to promote functional recovery and optimize patient outcomes. However, variations in the utilization of specific modalities suggest opportunities for further exploration and standardization of practices to ensure consistent and evidence-based care delivery in the ICU. Moreover, the emphasis on patient and caregiver involvement underscores the importance of collaborative care models in promoting optimal outcomes for ICU patients.

### Clinical applications and future research directions

The results of the present study on the current rehabilitation practice in the ICU in UAE provide valuable insights into physical therapists’ views and their implications for clinical care and future research directions.

In adult critical care settings, the widespread adoption of essential physiotherapy interventions such as passive/active range of motion exercises, bed mobility exercises, and assistance in bed transfers highlights their importance in promoting functional mobility, preventing complications associated with immobility, and facilitating early rehabilitation in critically ill patients. These interventions play a crucial role in optimizing patient outcomes, reducing the risk of complications such as muscle weakness, contractures, and pressure ulcers, and improving overall functional recovery ( Hashem et al. 2016).

Moreover, the utilization of advanced interventions such as NMES and positioning to support ventilation optimization highlights the evolving role of physiotherapists in critical care settings. These interventions offer promising benefits in improving respiratory function, facilitating weaning from mechanical ventilation, and enhancing patient outcomes in the ICU ( Nonoyama et al. 2022). Therefore, healthcare providers should prioritize integrating evidence-based physiotherapy interventions into multidisciplinary ICU care protocols to optimize patient care and improve long-term outcomes.

Furthermore, the findings on using physiotherapy techniques for neonates in the NICU underscore the importance of early and proactive interventions in promoting neonatal development and optimizing outcomes in this vulnerable population. Passive range of motion exercises, therapeutic handling, and orofacial stimulation are critical in fostering neuromuscular development, facilitating oral feeding skills, and supporting sensory development in neonates ( Chokshi et al. 2013). Additionally, parental involvement and education, particularly in the context of kangaroo mother care, are essential for promoting parental bonding, empowerment, and involvement in neonatal care ( Chan et al. 2016).

Looking forward, future research should focus on several key areas to enhance the evidence base and advance the field of physiotherapy in critical care and neonatal care settings. Firstly, studies investigating the efficacy and optimal timing, duration, and frequency of physiotherapy interventions in critically ill patients are warranted to inform evidence-based practice guidelines and enhance the quality of care provided in the ICU. Additionally, research examining the long-term effects of physiotherapy interventions on patient outcomes, including functional recovery, quality of life, and healthcare utilization, can provide valuable insights into the impact of these interventions on patient care and healthcare resource utilization.

In neonatal care settings, future research should explore the efficacy and safety of emerging interventions such as hydrotherapy and taping techniques in promoting neonatal development and optimizing outcomes in the NICU. Furthermore, studies investigating the effects of parental involvement and education on neonatal outcomes, family satisfaction, and healthcare utilization can inform the development of family-centred care models and support programs aimed at promoting parental bonding, involvement, and empowerment in neonatal care ( Jahan et al. 2021).

By addressing these research priorities and embracing innovative approaches, healthcare providers can enhance the quality of care provided to critically ill patients and neonates in intensive care settings, optimize patient outcomes, and improve the overall delivery of physiotherapy services in clinical practice.

### Study Limitations

While the study provides valuable insights into applying physiotherapy techniques in critical care and neonatal settings, some limitations should be acknowledged. First, the reliance on a convenience sample of physiotherapists introduces potential sampling bias. This sampling method may not adequately represent all practitioners in critical care and neonatal settings, potentially skewing the findings toward individuals with specific interests or expertise in physiotherapy. Consequently, the results may not generalize to the broader population of physiotherapists working in diverse healthcare contexts. Second, the data collected were based on self-reported responses from physiotherapists, which may be susceptible to self-reporting bias. Participants may have provided responses influenced by recall or social desirability bias, leading to overestimating, or underestimating certain practices. Moreover, self-reported data may not accurately reflect actual clinical practices, as respondents may have provided responses, they deemed more socially acceptable or aligned with best practices. Lastly, the study’s cross-sectional design only provides a snapshot of practices at a single point in time. While cross-sectional studies offer valuable insights into current practices, they do not allow for examining temporal trends or establishing causal relationships. Longitudinal studies would be necessary to assess changes in physiotherapy practices over time and their potential impact on patient outcomes.

## Conclusion

In conclusion, this study provides valuable insights into using physiotherapy techniques in critical care and neonatal settings. The findings highlight the widespread adoption of essential interventions such as passive range of motion exercises, positioning techniques, and therapeutic handling in promoting functional mobility and optimizing outcomes for patients in intensive care units (ICUs) and neonatal intensive care units (NICUs). Moreover, the study underscores the evolving role of physiotherapists in critical care settings, with interventions such as neuromuscular electrical stimulation and ventilation optimization techniques increasingly utilized to enhance patient outcomes. Future efforts should focus on standardizing physiotherapy protocols across different institutions and healthcare settings to ensure consistency and quality of care delivery.

## Ethical considerations

This study was conducted in full compliance with ethical guidelines and principles for research involving human participants. Ethical approval for this research project was obtained from the Ethics Committee of the Office of the Vice Chancellor for Research & Graduate Studies at the University of Sharjah, UAE. The project was approved under reference number REC-21-07-04-01-S on 06/07/2021.

After obtaining the approval from the ethical committee, all participants signed a written informed consent prior to their involvement in the study. Confidentiality and anonymity of participants were maintained throughout the research process. All data collected were securely stored and used solely for the purposes of this study. Participants were informed of their right to withdraw from the study at any time without any repercussions.

The research adhered to the ethical standards outlined in the Declaration of Helsinki and other applicable international and institutional ethical guidelines.

## Data Availability

The data generated and analyzed during this study are not publicly available due to ethical and confidentiality considerations, as outlined by the Ethics Committee of the Office of the Vice Chancellor for Research & Graduate Studies at the University of Sharjah, UAE. Participant data contain sensitive personal information, and sharing such data publicly could compromise confidentiality and anonymity. The Institutional Review Board (IRB) has mandated that data sharing is permissible only under specific conditions that ensure participant privacy and align with ethical guidelines. Access to the data may be granted to qualified researchers for legitimate academic purposes upon request. Requests for access must be submitted in writing to the corresponding author through fhegazy@sharjah.ac.ae. Applicants are required to provide a detailed research proposal outlining the purpose of their request and how the data will be used. Additionally, applicants must agree to adhere to strict confidentiality agreements and institutional guidelines regarding data handling. Access will be granted at the discretion of the Ethics Committee, subject to the signing of a data use agreement.
