# Supplementary material for: Current Rehabilitation Practices of Physiotherapists in Intensive Care Units in the UAE: A National Survey
Source: F1000Res. 2025 Jan 21;14:117. [Version 1] doi: 10.12688/f1000research.159853.1 (PMC12006783; doi:10.12688/f1000research.159853.1)
Supplement: Peer review history

# Reviewer report: Reviewer response for version 1

**DOI:** 10.5256/f1000research.175639.r375222

**Published:** 2025-04-17

**Authors:** Mohamed Khallaf

**Affiliations:** 1 University of st. Augustine for health sciences, Austin, USA

The study is well-organized, clear, and structured for an academic audience with strengths including: 

- **Clear Purpose & Scope**: You lay out your objectives and aims concisely, which anchors the rest of the paper well.
- **Contextual Framing**: The justification for the study is sound and taps into regional relevance (UAE, ICU/NICU rehab needs).

I have a few comments:

**Introduction**:

 - The United Arab Emirates (UAE) currently has 3,567 practicing physiotherapists, yet only 314 are registered members of the UAE chapter of the World Confederation for Physical Therapy (WCPT) ( AlKetbi et al. 2021). This sentence is repeated twice.   

**Survey development**: 

 No information is provided on whether the questionnaire was **translated**, especially if some participants were non-English speakers.

 Questionnaire Validity and Reliability:

- It’s not clear **how many experts** were involved in the validation process or what their **qualifications** and **experience levels** were.
- There’s no mention of whether there was a **consensus method** used (e.g., Delphi technique, inter-rater agreement). 
- **Suggestion**: Clarify the number, qualifications, and selection criteria of the experts.
- Discussion :The discussion does not fully explore **why gaps exist in practice** — e.g., low use of outcome measures or underutilization of certain interventions. 

Is the work clearly and accurately presented and does it cite the current literature?

Yes

If applicable, is the statistical analysis and its interpretation appropriate?

I cannot comment. A qualified statistician is required.

Are all the source data underlying the results available to ensure full reproducibility?

Yes

Is the study design appropriate and is the work technically sound?

Yes

Are the conclusions drawn adequately supported by the results?

Yes

Are sufficient details of methods and analysis provided to allow replication by others?

Yes

Reviewer Expertise:

Neurorehabilitation

I confirm that I have read this submission and believe that I have an appropriate level of expertise to confirm that it is of an acceptable scientific standard.

# Reviewer report: Reviewer response for version 1

**DOI:** 10.5256/f1000research.175639.r375223

**Published:** 2025-04-14

**Authors:** Dr.Omnya Samy Abdallah Ghoneim

**Affiliations:** 1 Department of Physical Therapy for Pediatrics and Pediatric Surgery, Faculty of Physical Therapy, Badr University in Cairo, Cairo, Egypt

Type: Cross-sectional survey-based research

 Objective: To investigate the current physiotherapy practices in ICU settings across the UAE and evaluate their alignment with evidence-based guidelines.

 The study addresses a timely and critical issue in rehabilitation: the variation and standardization of ICU physiotherapy practices in the UAE. It fills a significant research gap, particularly in a region with limited published data on ICU physiotherapy practices.

 The methodology is sound, and the discussion is well contextualized, though larger, more inclusive studies and mixed-method approaches would strengthen generalizability and depth.

 Ethical approval and clear consent processes.

 All the source data underlying the results is available through written requests to the corresponding author.

Is the work clearly and accurately presented and does it cite the current literature?

Yes

If applicable, is the statistical analysis and its interpretation appropriate?

Yes

Are all the source data underlying the results available to ensure full reproducibility?

No

Is the study design appropriate and is the work technically sound?

Yes

Are the conclusions drawn adequately supported by the results?

Yes

Are sufficient details of methods and analysis provided to allow replication by others?

Yes

Reviewer Expertise:

Pediatric rehabilitation, Sensory integration, occupational therapy.

I confirm that I have read this submission and believe that I have an appropriate level of expertise to confirm that it is of an acceptable scientific standard.

# Reviewer report: Reviewer response for version 1

**DOI:** 10.5256/f1000research.175639.r361737

**Published:** 2025-02-18

**Authors:** Leda Tomiko Yamada da Silveira

**Affiliations:** 1 University of Sao Paulo, University Hospital, Sao Paulo, Brazil

Dear authors,

 I appreciate the opportunity to revise this manuscript. I believe that mapping the Physiotherapy practice panel in a country is an essential step to assist in the development of plans that allow the improvement and growth of the profession and also to capture and register regional data that may be important in the global vision of Physiotherapy. However, I believe the manuscript needs improvement. Please find below a few comments and suggestions to the authors.

**Abstract**

1. There is contents belonging to the ‘Results’ presented in the ‘Methods’ section of the Abstract. The ‘Methods’ section should mention important information such as the eligibility criteria, questionnaire delivery process, data analysis plan.
2. The ‘Results’ that are presented in the abstract, such as the most and least performed procedures, do not give the readers the idea of variety of practices among ICU, which seems to be the main findings (at least, this is what is stated in the ‘Conclusion’). I suggest rephrasing it to reflect the authors’ conclusions from the findings.
3. Please check spelling and grammar, such as in “ *in-activity*”, “could cause diaphragmatic weakness *and which* is considered one of the most *life threatening* ICU complications”, “a UK study was conducted to investigate *the use of physiotherapists of evidence-based practice”.* Also, the fluidity of the text can be improved. The manuscript would benefit from an English language check.

**Introduction**

1. In the sentence: “For example, prolonged admission to the ICU could lead to physical in-activity and pressure ulcers which potentially cause muscular atrophy, generalized weakness sepsis, infections, and deep vein thrombosis.”, it looks like the pressure ulcer can cause muscle atrophy etc etc, and I don’t believe this is what was meant. Perhaps the authors should rephrase this statement.
2. The sentence “These include Medical ICUs for severe non-surgical conditions, Surgical ICUs for post-surgery recovery, Cardiac Care Units for critical heart issues, and specialized units like Neonatal ICUs for newborns, Pediatric ICUs for children, Neuro-ICUs for brain and nervous system conditions, Trauma ICUs, Burn Units, Respiratory ICUs, and Transplant ICUs” could be shortened; there is no need to be so specific. For example, there is no need to say that pediatric ICU is meant for children.
3. In the paragraph that starts with “Several studies have reported the rehabilitation practice of physiotherapists in various countries.”, the references that follow this sentence are not appropriated to base that affirmation. I suggest revising the choice of references in this paragraph.

A few examples (not mandatory) that authors could use or consult in order to find other references are refer 1 to 7.

 4. The fifth and sixth paragraphs of the ‘Introduction’ section have some repeated information, even a repeated full sentence. I suggest rewriting both paragraphs into one single well-structured paragraph.

**Study objectives**

1. The study objective is simply: to explore the current physiotherapy practices in ICUs across the UAE.

The other sentences presented in this section (to improve patient outcomes and elevate the quality of care, to inform healthcare policies optimizing resource allocation etc etc) is not what the study will be able to answer, therefore, they should be removed from this section. They can be placed in the end of the ‘Introduction’, in order to explain the reasons for which this study is important.

 The objective of a study should describe solely what the authors intend to do and what the findings from the proposed methodology can answer.

**Methods**

1. Regarding the study design, this is study is simply a cross-sectional study. Not prospective. A cross-sectional study cannot be prospective. A longitudinal study can be prospective or retrospective, because investigators follow participants through time. But in a cross-sectional study the participants are approached only once, there is no time sequence.
2. Could the authors please be more specific about the sample size calculation? For example, which outcome from the cited reference was used to calculate the sample size? What is this 90% proportion the authors mention?
3. In the ‘Statistical analysis’ section, the word ‘correlation’ is used incorrectly. The word ‘association’ is more appropriate in this case.  Correlation can only be calculated for two quantitative variables.

**Results**

1. The presentation of Table 1 is not usual. It is more common to enumerate the variables on the left column (vertically), and n (%) are usually presented in the same column, by using parenthesis. Also, all abbreviations must be explained in the Table’s legend.
2. The results must contain exclusively the study’s results. There are some phrases that should move to ‘Discusson’; basically, phrases that describe some kind of judgement or reasoning about the results, such as (but not limited to): “(…) suggesting potential variability in clinical protocols.”; “suggesting potential differences in practice patterns among physiotherapists.”, “This suggests that nebulizer therapy is commonly integrated into respiratory treatment plans, although there may be variations in timing and protocol adherence.”, “highlighting potential inconsistencies in patient education practices.”
3. Tables 2 and 3 need to explain the format in which the results are presented, i.e., absolute and relative numbers [n(%)].
4. Since Table 4 mentions the work in the neonatal ICU, I think it is necessary to identify that the other answers (Tables 2 and 3) refer to the work in the adult ICU. Also, I suggest mentioning in the ‘Methods’ that physiotherapists that worked in adults or neonatal ICUs were invited to answer the survey.
5. Results presented in the text of the ‘Results’ section is repeating much of the information in the Tables.
6. Authors did not report the percentage of responses they got from their survey (response rate), which is an important parameter in survey students since they may have selection bias.]
7. The meaning for the abbreviation NICU is not informed in the text.

**Discussion**

1. The ‘Discussion’ section is well-written and provides insights from the study’s results. However, I believe it is too long and shortening the text would be helpful.

**Conclusion**

1. Intensive care units (ICUs) and neonatal intensive care units (NICUs): these words were used before in the text, therefore, the abbreviation meaning should have already been informed to readers.

Is the work clearly and accurately presented and does it cite the current literature?

Yes

If applicable, is the statistical analysis and its interpretation appropriate?

Partly

Are all the source data underlying the results available to ensure full reproducibility?

No

Is the study design appropriate and is the work technically sound?

Partly

Are the conclusions drawn adequately supported by the results?

Partly

Are sufficient details of methods and analysis provided to allow replication by others?

Partly

Reviewer Expertise:

Physiotherapy applied at the Intensive Care Unit; Physiotherapy applied to in-hospital patients

I confirm that I have read this submission and believe that I have an appropriate level of expertise to state that I do not consider it to be of an acceptable scientific standard, for reasons outlined above.

## References

[1] : The scope of physiotherapy services provided in public ICUs in Greece: A pilot study. *Physiother Theory Pract* .2017;33(2) : 10.1080/09593985.2016.1266718 138-146 10.1080/09593985.2016.1266718 28075178

[2] : National Profile of Physical Therapists in Critical Care Units of Sri Lanka: Lower Middle-Income Country. *Phys Ther* .2016;96(7) : 10.2522/ptj.20150363 933-9 10.2522/ptj.20150363 26893503

[3] : Mobilization practices in the ICU: A nationwide 1-day point- prevalence study in Brazil. *PLoS One* .2020;15(4) : 10.1371/journal.pone.0230971 e0230971 10.1371/journal.pone.0230971 32240249 PMC7117707

[4] : Rehabilitation and exercise prescription in Australian intensive care units. *Physiotherapy* .2008;94(3) : 10.1016/j.physio.2007.11.004 220-229 10.1016/j.physio.2007.11.004

[5] : A profile of European intensive care unit physiotherapists. European Society of Intensive Care Medicine. *Intensive Care Med* .2000;26(7) : 10.1007/s001340051292 988-94 10.1007/s001340051292 10990117

[6] : Physical therapy in the intensive care unit: A cross-sectional study of three Asian countries. *PLoS One* .2023;18(11) : 10.1371/journal.pone.0289876 e0289876 10.1371/journal.pone.0289876 37943762 PMC10635439

[7] : Definición del rol y las competencias del kinesiólogo en la unidad de cuidados intensivos.Rev Arg de Ter Int. 2018;
